# Estimation of Reduction in Influenza Vaccine Effectiveness Due to Egg-Adaptation Changes—Systematic Literature Review and Expert Consensus

**DOI:** 10.3390/vaccines9111255

**Published:** 2021-10-29

**Authors:** Raul Ortiz de Lejarazu-Leonardo, Emanuele Montomoli, Radek Wojcik, Solomon Christopher, Anne Mosnier, Elena Pariani, Antoni Trilla Garcia, Helmut Fickenscher, Barbara C. Gärtner, Ravi Jandhyala, Maria Zambon, Catherine Moore

**Affiliations:** 1 Valladolid National Influenza Centre, Calle Rondilla de Santa Teresa s/n, 47009 Valladolid, Spain; lejarazu@gmail.com; 2 Department of Molecular Medicine, University of Siena, 53100 Siena, Italy; montomoli@vismederi.com; 3 Medialis Ltd., Banbury OX16 0AH, UK; solomon@medialis.co.uk (S.C.); ravi@medialis.co.uk (R.J.); 4 Open Rome, 75008 Paris, France; amos@openrome.org; 5 Department of Biomedical Science for Health, University of Milan, 20122 Milan, Italy; Elena.Pariani@unimi.it; 6 Preventive Medicine and Epidemiology, Hospital Clínic, University of Barcelona, 08007 Barcelona, Spain; ATRILLA@clinic.cat; 7 Institute for Infection Medicine, Kiel University, 24118 Kiel, Germany; fickenscher@infmed.uni-kiel.de; 8 University Medical Center Schleswig-Holstein, 24105 Kiel, Germany; 9 Institute for Microbiology and Hygiene, Saarland University, Faculty of Medicine and Medical Center, Building 43, 66421 Homburg/Saar, Germany; barbara.gaertner@uks.eu; 10 Public Health England, London SE1 8UG, UK; maria.zambon@phe.gov.uk; 11 Wales Specialist Virology Centre, Public Health Wales, Cardiff CF14 4XW, UK; catherine.moore2@wales.nhs.uk

**Keywords:** influenza, vaccination, egg adaptations, antigenic, drift, effectiveness

## Abstract

Background: Influenza vaccines are the main tool to prevent morbidity and mortality of the disease; however, egg adaptations associated with the choice of the manufacturing process may reduce their effectiveness. This study aimed to estimate the impact of egg adaptations and antigenic drift on the effectiveness of trivalent (TIV) and quadrivalent (QIV) influenza vaccines. Methods: Nine experts in influenza virology were recruited into a Delphi-style exercise. In the first round, the experts were asked to answer questions on the impact of antigenic drift and egg adaptations on vaccine match (VM) and influenza vaccine effectiveness (IVE). In the second round, the experts were presented with the data from a systematic literature review on the same subject and aggregated experts’ responses to round one questions. The experts were asked to review and confirm or amend their responses before the final summary statistics were calculated. Results: The experts estimated that, across Europe, the egg adaptations reduce, on average, VM to circulating viruses by 7–21% and reduce IVE by 4–16%. According to the experts, antigenic drift results in a similar impact on VM (8–24%) and IVE (5–20%). The highest reduction in IVE was estimated for the influenza virus A(H3N2) subtype for the under 65 age group. When asked about the frequency of the phenomena, the experts indicated that, on average, between the 2014 and 19 seasons, egg adaptation and antigenic drift were significant enough to impact IVE that occurred in two and three out of five seasons, respectively. They also agreed that this pattern is likely to reoccur in future seasons. Conclusions: Expert estimates suggest there is a potential for 9% on average (weighted average of “All strains” over three age groups adjusted by population size) and up to a 16% increase in IVE (against A(H3N2), the <65 age group) if egg adaptations that arise when employing the traditional egg-based manufacturing process are avoided.

## 1. Introduction

Seasonal influenza is an acute respiratory disease associated with significant morbidity and mortality worldwide [1,2]. Global annual influenza infection rates range between 10 and 30% [3], with an estimated 250,000 to 500,000 deaths worldwide due to flu-associated complications [4]. The European Centre for Disease Prevention and Control (ECDC) has estimated that, in the European Union alone, each year there are between 4 million and 50 million symptomatic cases, of which 15,000–70,000 result in death [5]. Influenza annually causes an estimated 6–14 billion € burden in Europe alone [6].

Seasonal vaccination is the most effective strategy for the prevention of influenza-associated outcomes [3]. Yet, vaccine effectiveness (VE)—the protection conferred by vaccination in real-world settings—for influenza remains suboptimal [7,8]. Observational studies have further demonstrated that influenza vaccine effectiveness (IVE) varies across influenza (sub)types [9] and is lower for influenza A(H3N2) compared to influenza A(H1N1) and influenza B viruses [10]. A systematic review of IVE between the 2004 and 2015 influenza seasons in the United States estimated the pooled VE for A(H1N1pdm09) to be 61% (95% CI: 57–65%) and 54% (95% CI: 46–61%) for matched influenza type B compared to only 33% (95% CI: 26–39%) for A(H3N2) [10]. This is of particular importance as seasons wherein A(H3N2) circulation predominates are associated with a higher morbidity and excess mortality compared to other influenza strains [11,12].

Multiple factors can affect the performance of influenza vaccines, including the characteristics of the vaccinated population (such as health condition, age, prior influenza exposure, and gender); influenza virus subtype; and vaccine match, a key predictor of IVE [8,9,13,14]. Low IVE is historically discussed in the context of antigenic mismatch between the vaccine and circulation strains due to antigenic drift caused by immune selective pressure [15,16,17]. Despite being a similar phenomenon, egg adaptation changes of influenza virus have been known since the 1940′s [18], only recently gaining substantial from the scientific community. This form of drift caused by selective pressure that arises due to virus propagation in eggs is increasingly recognised as a significant cause of antigenic mismatch of influenza vaccine viruses [7]. Therefore, egg-based platforms, mainly used in the manufacturing of influenza vaccines, may play a significant role in contributing to the antigenic mismatch with circulating flu viruses [7,19].

The production of influenza vaccines is conditioned by the continuous antigenic drift in flu viruses. This necessitates annual updates to retain match against circulating strains in a multi-step process. Global surveillance of influenza viruses is carried out by the Global Influenza Surveillance and Response System (GISRS). World Health Organization (WHO) Collaborating Centres perform the genetic and antigenic characterisation of candidate vaccine viruses (CVV) based on the results from the GISRS. Finally, the WHO reconvenes every February and September to determine the composition of flu vaccines for the next season in the northern and southern hemispheres. Following a reassortment of candidate wild viruses with strains that have improved yield in eggs, the new CVV is shared with manufacturers for vaccine preparation [7].

Currently, the majority of influenza vaccines are produced using embryonated hen’s eggs, primarily due to supply availability, low costs, and historical use [3,7]. However, differences between the avian and human sialic acid composition at the cell surface, which act as the receptor for influenza viruses binding and mediates viral cell entry, drive the selection for viral variants containing mutations that are better adapted for propagation in eggs [20]. These egg-adaptive substitutions enhance binding affinity and avidity to avian cells. Unfortunately, they may also impact virus antigenicity and have important implications with regards to immune priming by seasonal flu vaccination [21,22] and the subsequent effectiveness of the influenza vaccine [23]. Certainly, egg adaptation changes have been linked to a reduction in IVE (mainly against A(H3N2)) in some of the previous influenza seasons. Skowronski et al. demonstrated that low IVE in 2012 to 2013 was associated with mutations in the egg-adapted A(H3N2) vaccine viruses [16]. Egg adaptation changes have been implicated in the poor performance of the vaccine against A(H3N2) viruses during the influenza seasons of 2014 to 2015, 2016 to 2017, and 2017 to 2018 [15,16,24,25,26]. The proportion of the overall reduction of IVE contributed by the egg-based manufactured process is, therefore, a valuable piece of information to guide future manufacturing decisions. While virus antigenic drift cannot be controlled, egg adaptation changes may be potentially removed by the selection of technology that does not involve avian cells.

Nonetheless, the impact of this particular phenomenon on vaccine effectiveness remains to be estimated in the real-world. Studies comparing relative vaccine effectiveness (rVE) between cell- and egg-based vaccines have been conducted before, pointing to a better performance of cell-based vaccines against A(H3N2). However, the results remain inconclusive with regards to real estimations on the reduction of vaccine effectiveness due to egg adaptation changes [27,28,29,30]. The objective of this study was to quantify the impact of egg adaptations changes on the effectiveness of traditional trivalent and quadrivalent influenza vaccines.

## 2. Methods

The study was conducted utilising the Delphi-type technique to generate group consensus between July and December 2020. The procedure involved a two-stage survey of expert opinion on the impact of antigenic drift and egg adaptation changes on vaccine match (VM) and effectiveness (IVE) and their frequency between the 2014 and 2019 influenza seasons in the EU5 countries. These data were complemented with two systematic literature reviews focused on influenza VM and IVE (for the identical period).

### 2.1. Systematic Literature Reviews

Two literature reviews were performed, which followed the Preferred Reporting Items for Systematic Reviews and Meta-Analyses (PRISMA) statement [31]. A Systematic Literature Review (SLR) was conducted for the research question “Influenza vaccine match (TIVs (Trivalent Influenza Vaccines)) and QIVs (Quadrivalent Influenza Vaccines)) to circulating viruses between 2014–2019”. A second SLR addressed the research question “Effectiveness of TIV and QIV influenza vaccine in seasons 2014–2019”. The included articles reported European findings on IVE and VM, being original research, surveillance and sentinel data, systematic literature reviews and metanalyses, in English. Studies on non-egg-based vaccines, RCT’s, laboratory experiments, and non-European or non-human influenza viruses were excluded in the process of the SLR. The bibliographic searches were carried out using the PubMed database. Other public health websites, such as Eurosurveillance, I-MOVE, ECDC, WHO), Robert-Koch institute, GOV.UK, Sante publique France, Institut Pasteur/National Reference Center for Respiratory Viruses, Istituto Superiore di Sanità, and the Instituto de Salud Carlos III/Sistema de Vigilancia de la Gripe en España were also searched for relevant publications. Literature reviews of the search results were performed by two analysts and restricted to studies published in English, specific to influenza seasons between 2014/15 and 2018/19 and regarding only traditional TIV and QIV influenza vaccines. Additional reviews of publications provided by the experts to support responses provided in survey rounds one and two were completed systematically by a single analyst (a virologist). Data collected included influenza vaccine match or mismatch against circulating strains (including a percentage of match/mismatch, when presented), report of antigenic drift or egg adaptation changes, and measurement of vaccine effectiveness.

### 2.2. Assessment of Expert Opinion

Eleven European influenza experts from five European countries (France, Germany, Italy, Spain, and the UK) were invited to participate in the study via professional networks. The inclusion criteria for the experts were: background and expertise in Public Health, Epidemiology of influenza or Virology, and/or peer-review publications or membership to relevant organisations.

In the first survey round (using open-ended questions), experts were asked to provide a mean estimate of the increase of influenza VM and IVE in the absence of antigenic drift or egg adaptations. The responses of each expert were specific to their country and regarded only influenza seasons between 2014 and 2019. The experts were also asked to provide the frequency of the impact of these phenomena on vaccine match and effectiveness for the same period. The answers from all experts were processed using summary statistics to produce estimates at both the country level and European level. In the second survey round, experts were presented with both sets of estimates (country and European levels) and were allowed to revise their responses accordingly. Answers to the second survey were combined utilising summary statistics at country and EU5 level, as well as the EU5 averages estimated by experts for individual countries.

An estimated percentage increase in vaccine effectiveness was calculated as an absolute increase (IVE in the absence of antigenic drift, or egg adaptation IVE in the presence of both antigenic drift and egg adaptations). A percentage increase in VM was similarly calculated as an absolute increase. Summary statistics such as mean, and range (minimum, maximum) were used to present the average percentage increase.

## 3. Results

### 3.1. Participants

Of the eleven experts who agreed to take part in the study, nine participated. The group of nine experts included six virologists; two public health experts; and an epidemiologist physician recruited from five European countries (France, Germany, Italy, Spain, and the UK).

### 3.2. Systematic Literature Review

**Figure 1:**
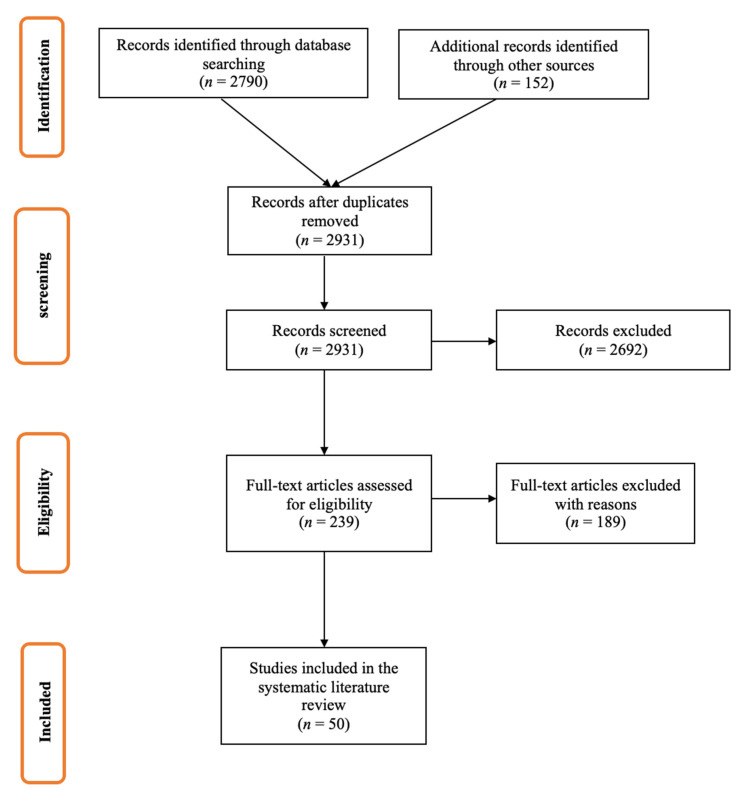
Literature review results for the research question: “Influenza vaccine match (TIVs and QIVs) to circulating viruses between 2014 and 2019”.

**Figure 2:**
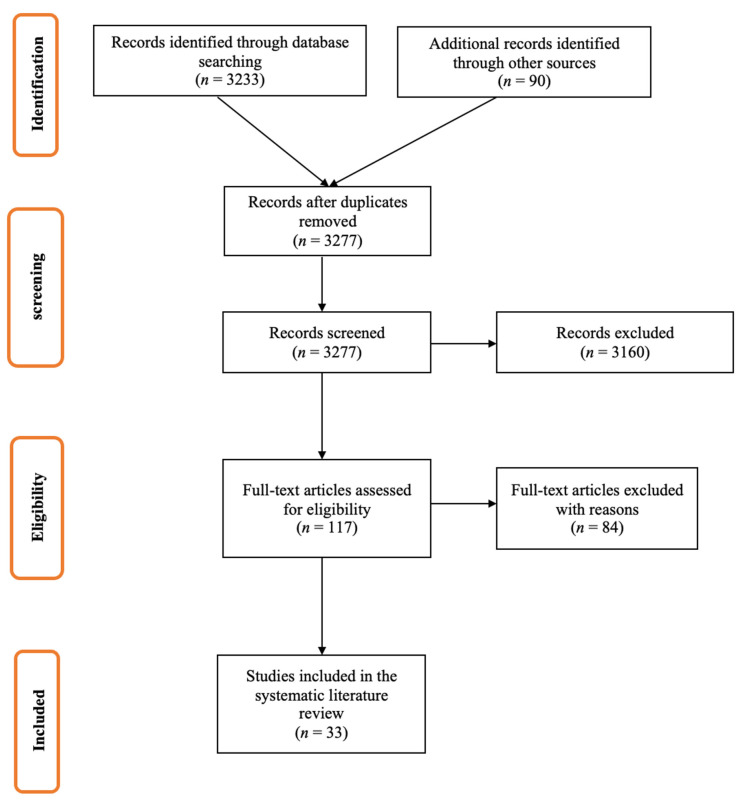
Literature review results for the research question: “Effectiveness of TIV and QIV influenza vaccines in seasons 2014–2019”.

The literature search of relevant databases and websites produced 6208 publications, which were screened for eligibility. Of these, 356 publications progressed for full-text review, and 83 were included in the systematic review (Figure 1 and Figure 2). Principal reasons for exclusion were data reported outside of the geographic scope (EU5 countries) and the inclusion of the wrong population, outcome, or study design.

### 3.3. Survey of Expert Opinion

All nine experts provided estimates for the impact of antigenic drift and egg adaptation changes on vaccine effectiveness and the frequency with which these were likely to have occurred between 2014 and 2019. The final estimates are presented in Table 1, Table 2, Table 3 and Table 4.

The study surveyed expert opinions on the EU5 and country-level estimates separately. The EU5 average increase in VM in the absence of egg adaptation changes was estimated to be between 7% and 21% (depending on the influenza virus strain, Table 1), which experts indicated was a strong predictor of vaccine effectiveness (in a distinct question of the survey) (Table 2). Furthermore, the mean reduction (9%) of IVE due to egg adaptation changes was estimated to be between 4% and 16%, also depending on influenza virus strain (Table 3, column e).

**Figure 3:**
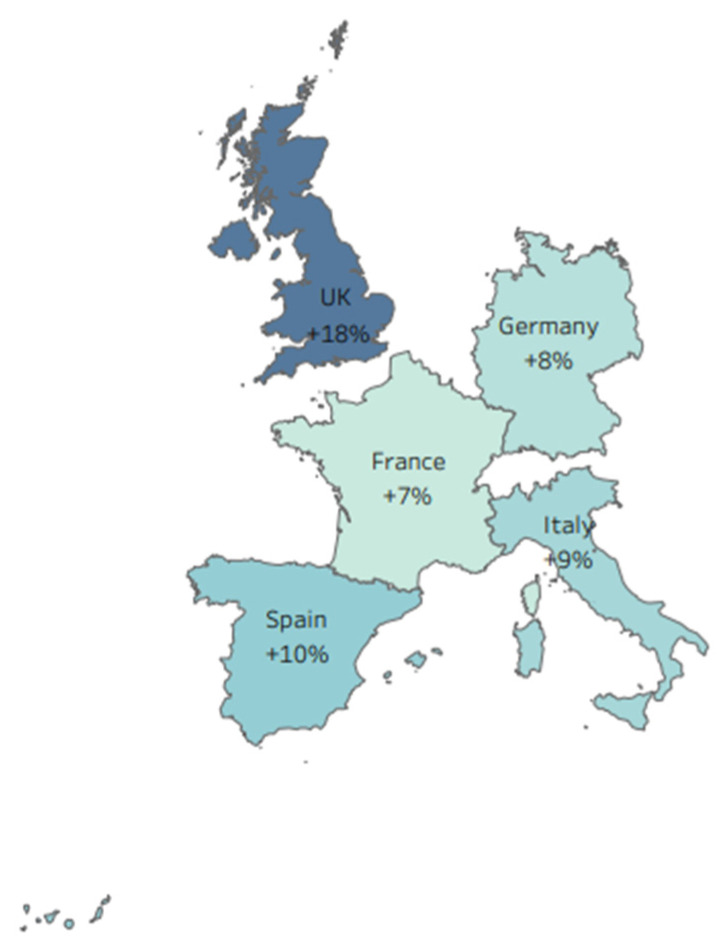
Mean estimates of the increase in IVE in the absence of egg adaptations for “all strains” per country (TIV and QIV only). The figure was developed using Tableau (https://www.tableau.com; accessed on 9 August 2021) based on data generated in this study and is a copyright of Medialis Limited.

**Figure 4:**
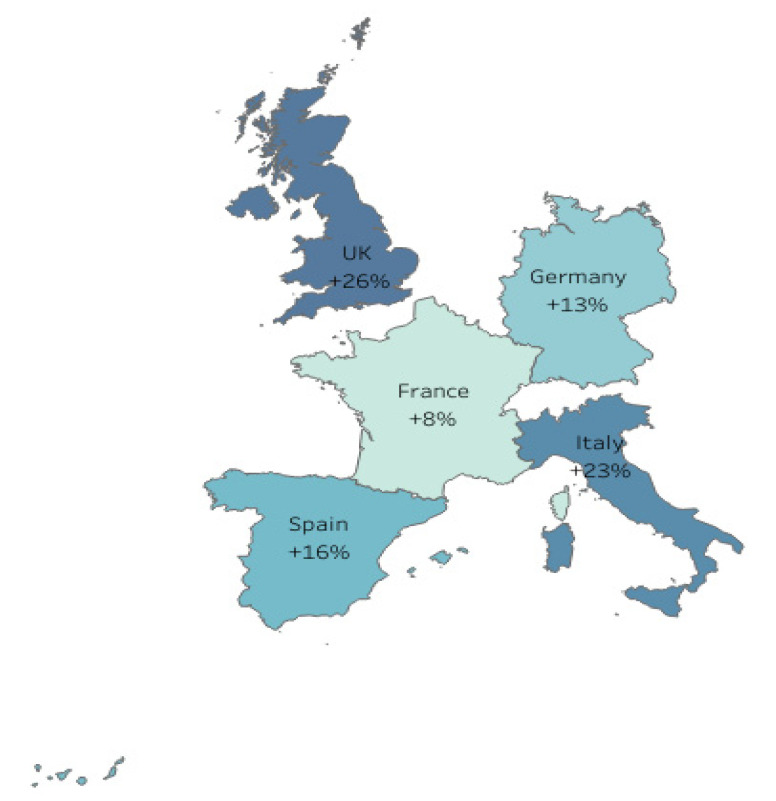
Mean estimates of the increase in IVE in the absence of egg adaptations for A(H3N2) per country (TIV and QIV only). The figure was developed using Tableau (https://www.tableau.com; accessed on 9 August 2021) based on data generated in this study and is a copyright of Medialis Limited.

The highest reduction in IVE was estimated for the A(H3N2) virus subtype and the 18–64 age group (up to 16%, see Table A1, Appendix A). A greater variation was observed in the estimates for individual countries, as opposed to estimates at the EU5-level (Figure 3 and Figure 4 and Table A2, Table A3 and Table A4, Appendix A). However, when the country-level estimates were averaged, they were very similar to the EU5-level estimates (Table 3).

According to the experts, antigenic drift resulted in a similar, however marginally higher, impact on VM (8–24%, Table 1) and IVE (5–20%, Table 3, column a).

When asked about the frequency of the phenomena, the experts indicated that, on average, between 2014 and 2019, egg adaptation changes and antigenic drifts significant enough to impact IVE occurred in two and three out of the five seasons, respectively. They also agreed that this pattern is likely to reoccur in future seasons.

## 4. Discussion

Egg adaptation substitutions that occur during vaccine manufacturing can impact the performance of flu vaccines and have been previously described to be implicated in the reduction of vaccine effectiveness [16,24]. However, the extent to which egg adaptation changes can affect IVE has not been measured, as indicated by the absence of publications reporting this particular outcome in the systematic literature review. This study is a first attempt at quantifying the impact that egg adaptation changes have on vaccine effectiveness. It is important to have these estimates, because they signal the scale of potential inefficiencies of traditional egg-based manufacturing technology, for which alternatives exist [32,33]. Antigenic drift was reviewed in this study alongside egg adaptations, as it is a very similar process and its impact on IVE has been established; however, notably it cannot be avoided, in contrast to the egg adaptations.

The impact of mutations, i.e., egg adaptation changes and antigenic drift, on IVE is difficult to measure with traditional epidemiological studies, conducted chiefly in real-world settings. In the absence of multicentric European-wide studies on IVE, which include genetic characterization of viral strains and supporting studies evaluating this specific research question, the use of consensus expert opinion in addressing this issue was deemed appropriate as it falls within a well-established tradition of generating legitimate evidence [34,35]. Despite the level of evidence (in the evidence pyramid) carrying an inherent degree of uncertainty, it was important to highlight this issue to point to the avoidable risk, relating to egg-adaptation changes, and the likely reduction in IVE. Thus, to answer the research question, prominent experts on virology and public health from five European countries were invited to participate in this study. Experts provided their estimates on the reduction of vaccine match and effectiveness caused by egg adaptations and antigenic drift, during the 2014–2019 influenza seasons. The questions were dedicated only to traditional (egg-based) TIV and QIV vaccines. Adjuvanted TIV/QIV vaccines, Live Attenuated Influenza Vaccines (LAIV), and cell-based or recombinant vaccines were not considered in this study.

**Figure 5:**
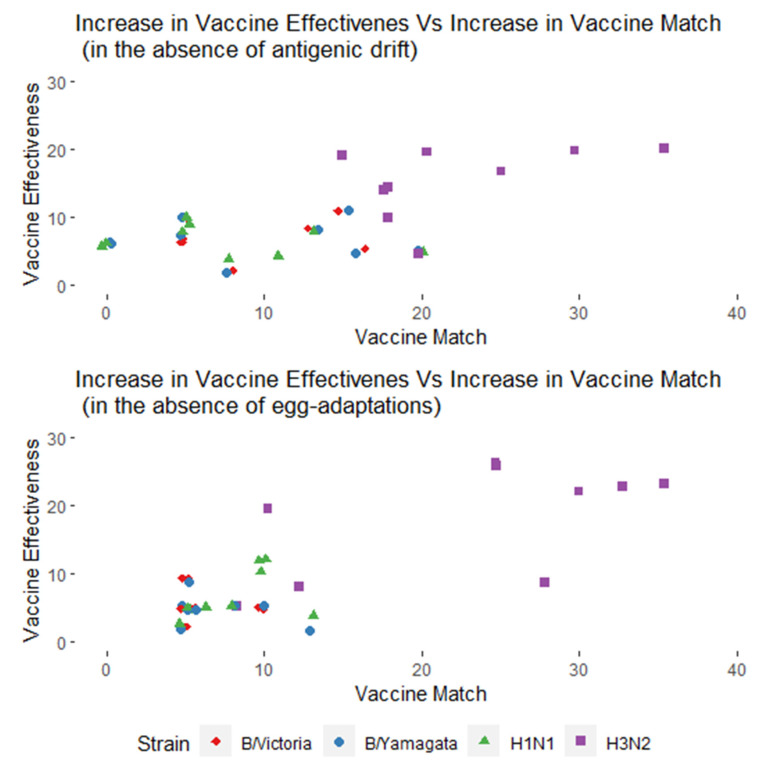
Correlation between vaccine effectiveness and vaccine match in the experts’ responses.

The study questions were constructed to ask about the direct consequence of virus evolution on the vaccine performance (VM) and IVE, which is the key endpoint; however, IVE is also influenced by several other factors. These two outcomes were also used to internally validate the relationship between VM and IVE within the experts’ answers. The results confirmed a positive correlation in line with the literature—an increase in VM is associated with increased IVE. This correlation was the strongest and proportionally higher for the A(H3N2) strain, compared to the other strains (Figure 5).

Examination of the selected publications from the literature review allowed for the extraction of estimates for VM and VE, mapped to influenza seasons when the antigenic drift or egg adaptation changes occurred, with the majority of the data being recorded for A(H1N1)/A(H1N1pdm09) and A(H3N2). Even so, near completeness of data for A(H1N1) and A(H3N2) was only available for Germany, Spain, and the United Kingdom. This was due, mainly, to estimates of IVE and VM from France and Italy in several studies being presented pooled together with data from European countries not included in this study. This data was presented to the experts within the survey. Analysis of the data collected from the SLR demonstrated that a vaccine match was mostly observed for A(H1N1) across all five European countries. Furthermore, A(H1N1) exhibited (with rare exceptions) the highest values for vaccine effectiveness across all seasons. Conversely, A(H3N2) vaccine viruses were mismatched against circulating strains in two or more seasons for the majority of the five countries, primarily due to antigenic drift. Vaccine effectiveness against A(H3N2) was also demonstrably lower compared to A(H1N1) and influenza B. Finally, quantification of the impact of egg adaptation changes on vaccine match or effectiveness was not described in any of the publications retrieved from the literature reviews.

The expert estimates of VM and IVE showed a similar pattern of being the most pronounced for the A(H3N2) subtype: the estimated increase in VM and IVE for A(H3N2) was two-fold compared to the increase in IVE for A(H1N1pdm09) or influenza B strains. These results are aligned with the current literature on this topic, in which the discussion of egg adaptation changes is predominantly conducted in the context of A(H3N2) [7]. The experts noted, however, that even within A(H3N2), the issue of virus variation affects different clades differently. While these issues affect the other strains to a lower degree, it is the A(H3N2) viruses, which are linked to the highest influenza-associated morbidity and mortality and have the poorest vaccine effectiveness among all strains [10,12,36] and have been the principal cause of burden from influenza disease in recent years [11].

Low vaccine effectiveness has been historically interpreted as a consequence of low VM to circulating strains due to ongoing antigenic drift [16]. The findings demonstrate that egg adaptation changes might be an equally important factor, contributing to the reduction of VM and IVE on a similar level as antigenic drift (while the German experts indicate the antigenic drift has a much greater impact). Where we observed expected variation in minimum and maximum responses, due to the high degree of uncertainty of the question itself, the average responses for the EU5 (as a level) and EU5 calculated from individual countries’ values were surprisingly similar, which supports the validity of these results (Table 3). Analysis of the results also points to children and adults under 65 suffer the most from the decrease in vaccine effectiveness due to egg adaptations. This population is also the most likely to cause an indirect cost burden, which is a key part of the total influenza burden [37,38]. If confirmed, under 65s may be the population that would benefit the most from the use of non-egg-based influenza vaccines.

This much needed work certainly broadens the discussion around the process of vaccine manufacturing and the impact it may have on IVE and public health. Although ongoing drift in circulating viruses cannot be controlled [16], egg adaptation substitutions, on the other hand, are dependent on the technology used for vaccine production and can be improved upon [16,39]. The adoption of alternative manufacturing techniques that do not include the propagation of influenza viruses in eggs can contribute to the improvement of vaccine effectiveness [10,16,28]. This study supports this assertion further by quantifying the increase in VE in the absence of egg-based manufacturing. Furthermore, manufacturing technologies continue to evolve in this area, most notably with the development of adjuvanted cell-based (aQIVc), recombinant (QIVr), mRNA, and most recently, self-amplifying-mRNA (sa-mRNA) vaccines. Of note, sa-mRNA vaccines have the potential to elicit stronger cellular responses and generate significantly higher antibody titres at the same dose level as mRNA [40].

There are, however, limitations to this study. This work is focused on only a few countries in Europe, and, as such, generated data that may be limited by its geographic scope. Other potential limitations of this study include its design (expert opinion) and the limited number of participants. Our approach was adopted due to the difficulty in employing clinical trials or observational studies to address this particular research question. The latter has also resulted in a lack of a higher level of evidence available to support our findings, compounded by the fact that literature reviews from this study were done mostly for publications in the English language. The comparison between experts’ estimates and the estimates from the systematic literature review on the contribution of egg adaptation changes or antigenic drift to the reduction of IVE and VM was not possible, as this outcome was not reported in any of the included publications. To the best of our knowledge, estimates of the reduction in vaccine effectiveness due to egg adaptation changes have not been published yet. It is possible that otherwise available information was not included in this work, due to that restriction. Furthermore, given the scope of this study on the impact of egg adaptation changes on traditional TIVs and QIVs-LAIV, adjuvanted TIV/QIV, high-dose (egg-based), cell-based and recombinant vaccines (non-egg-based) were not included in this research. Although the overall vaccine effectiveness is contributed to by all of the above, traditional inactivated TIVs and QIVs are still the most extensively used vaccines in influenza immunisation programs, indicative of the relevance of our findings.

## 5. Conclusions

In this study, European experts evaluated the impact that egg adaptation changes have on vaccine mismatch and vaccine effectiveness, based on their research and/or professional experience and surveillance evidence collected during recent flu seasons. Overall, expert estimates suggested there is a potential for up to a 16% increase in IVE (against H3N2 and <65 age group) and, on average, 9%^2^ (6–10%; all strains and age groups) if egg adaptation changes that arise when employing the traditional egg-based manufacturing process are avoided. There are multiple factors affecting influenza vaccine effectiveness to varying degrees. While many of them are difficult to control, egg adaptations can be eliminated by changing the technology of production. Given this, the selection of alternative technologies for vaccine production without the use of eggs may contribute to an increase in IVE during future influenza seasons.

## Figures and Tables

**Table 1:** Expert estimates of the absolute increase in vaccine match in the absence of antigenic drift or egg adaptation changes, per influenza (sub)type, at the EU5 level, between 2014 and 2019.

Influenza (Sub)Type	Mean Increase in VM in the Absence of AD		Mean Increase in VM in the Absence of EA	
	Mean Est. (%)	Min–Max (%)	Mean Est. (%)	Min–Max (%)
A(H3N2)	24	20–31	21	10–30
A(H1N1pdm)	8	8–11	8	0–8
B/Yamagata	10	5–18	7	0–7
B/Victoria	10	5–19	7	0–7
All strains	19	12–26	18	10–21

EU5 (UK, DE, FR, ITA, and ESP); VM (vaccine match); AD (antigenic drift); and EA (egg adaptation changes).

**Table 2:** Expert estimates of the absolute increase in A(H3N2) vaccine match in the absence of antigenic drift or egg adaptation changes, specific for each country, between 2014 and 2019.

Country	Mean Increase in VM in the Absence of AD (%)	Mean Increase in VM in the Absence of EA (%)
UK	19	25
ITA	30	34
FR	15	12
ESP	20	29
DE	24	9

EU5 (UK, DE, FR, ITA, and ESP); VM (vaccine match); AD (antigenic drift); and EA (egg adaptation changes).

**Table 3:** Comparison of experts’ mean estimates of the absolute increase in vaccine effectiveness in the absence of antigenic drift or egg adaptation changes, per influenza (sub)type and age group, at the EU5 level between 2014 and 2019.

Influenza (Sub)Type	Age Group	EU5 Level Mean Increase w/o AD		EU5 Country Avg. Mean Increase w/o AD		EU5 Level Mean Increase in w/o EA		EU5 Country Avg. Mean Increase w/o EA	
		Mean Est. (%)	Min–Max (%)	Mean Est. (%)	Min–Max (%)	Mean Est. (%)	Min–Max (%)	Mean Est. (%)	Min–Max (%)
		a	b	c	d	e	f	g	h
A(H3N2)	<18 year	20	6–22	21	6–30	15	5–25	18	5–30
	18–64 year	15	5–20	15	5–20	16	5–22	18	5–30
	≥65 year	17	2–40	14	2–20	12	5–20	13	5–23
A(H1N1pdm)	<18 year	9	7–9	9	5–12	7	4–8	7	3–15
	18–64 year	8	4–9	7	3–10	7	4–8	7	3–13
	≥65 year	5	1–7	6	1–9	5	2–5	5	2–10
B/Yamagata	<18 year	8	6–8	9	5–19	6	2–6	7	2–15
	18–64 year	6	1–6	7	1–10	5	2–5	5	2–8
	≥65 year	6	1–7	6	1–10	4	1–5	4	1–5
B/Victoria	<18 year	8	6–8	9	5–19	6	2–6	7	2–15
	18–64 year	6	1–6	7	1–10	5	2–5	5	2–8
	≥65 year	6	1–7	6	1–10	4	1–5	4	1–5
All strains	<18 year	10	5–13	10	5–17	8	5–12	10	5–15
	18–64 year	10	5–10	10	5–15	10	5–14	12	5–23
	≥65 year	9	5–10	9	5–13	6	2–7	7	2–13

EU5 (UK, DE, FR, ITA, and ESP); VE (vaccine effectiveness); AD (antigenic drift); and EA (egg adaptation changes). Emboldened numerals are referenced and discussed in text.

**Table 4:** Experts’ mean estimates of the absolute increase in A(H3N2) vaccine effectiveness in the absence of antigenic drift or egg adaptation changes for each country between 2014 and 2019.

Country	Age Group	Mean Increase in VE in the Absence of AD (%)	Mean Increase in VE in the Absence of EA (%)
UK	<18 year	+30	+30
	18–64 year	+10	+30
	≥65 year	+10	+10
ITA	<18 year	+23	+26
	18–64 year	+18	+23
	≥65 year	+18	+21
FR	<18 year	+20	+8
	18–64 year	+20	+8
	≥65 year	+15	+8
ESP	<18 year	+15	+14
	18–64 year	+13	+17
	≥65 year	+10	+14
DE	<18 year	+15	+13
	18–64 year	+15	+13
	≥65 year	+15	+13
EU5	<18 year	+20	+15
	18–64 year	+15	+16
	≥65 year	+17	+12

EU5 (UK, DE, FR, ITA, and ESP); VE (vaccine effectiveness); AD (antigenic drift); and EA (egg adaptation changes).

## Data Availability

Not applicable.

## Appendix A

**Table A1:** Country-level and EU5-level survey responses on the IVE increase without egg adaptations.

Influenza (Sub)Type	Age Group	The Average Increase in Influenza Vaccine Effectiveness without Egg Adaptations (%)					
		ES	IT	DE	UK	FR	EU5 CAT
A(H3N2)	<18 year	14	26	13	30	8	15
	18–64 year	17	23	13	30	8	16
	≥65 year	14	21	13	10	8	12
A(H1N1pdm)	<18 year	7	5	5	15	5	7
	18–64 year	7	4	5	13	5	7
	≥65 year	6	4	5	3	5	5
B/Yamagata	<18 year	4	5	5	15	5	6
	18–64 year	4	4	5	8	5	5
	≥65 year	3	3	5	3	5	4
B/Victoria	<18 year	4	5	5	15	5	6
	18–64 year	4	4	5	8	5	5
	≥65 year	3	3	5	3	5	4
All strains	<18 year	8	11	8	15	7	8
	18–64 year	12	9	8	23	7	10
	≥65 year	8	9	8	3	7	6

EU5 (UK, DE, FR, ITA, and ESP) and IVE (influenza vaccine effectiveness).

**Table A2:** Country-level and EU5-level survey responses on the IVE increase without antigenic drift.

Influenza (sub)Type	Age Group	The Average Increase in Influenza Vaccine Effectiveness without Antigenic Drift (%)					
		ES	IT	DE	UK	FR	EU5 CAT
A(H3N2)	<18 year	15	23	15	30	20	20
	18–64 year	13	18	15	10	20	15
	≥65 year	10	18	15	10	15	17
A(H1N1pdm)	>18 year	8	10	7	10	10	9
	18–64 year	4	9	7	5	10	8
	≥65 year	2	9	7	5	7	5
B/Yamagata	>18 year	7	13	7	10	10	8
	18–64 year	3	9	7	5	10	6
	≥65 year	2	8	7	5	10	6
B/Victoria	<18 year	7	13	7	10	10	8
	18–64 year	3	9	7	5	10	6
	≥65 year	2	8	7	5	10	6
All strains	<18 year	7	15	10	10	10	10
	18–64 year	10	13	10	5	10	10
	≥65 year	9	13	10	5	10	9

EU5 (UK, DE, FR, ITA, and ESP) and IVE (influenza vaccine effectiveness).

**Table A3:** Country-level and EU5-level survey responses on the influenza VM increase without antigenic drift.

Influenza (Sub)Type	The Average Increase in Vaccine Match without Antigenic Drift (%)					
	ES	IT	DE	UK	FR	EU5 CAT
A(H3N2)	20	30	24	19	15	24
A(H1N1pdm)	10	5	17	0	5	8
B/Yamagata	12	10	17	0	5	10
B/Victoria	12	10	17	5	5	10
All strains	20	19	17	19	15	19

EU5 (UK, DE, FR, ITA, and ESP) and VM (vaccine match).

**Table A4:** Country-level and EU5-level survey responses on the influenza VM increase without egg adaptations.

Influenza (Sub)Type	The Average Increase in Vaccine Match without Egg Adaptations (%)					
	ES	IT	DE	UK	FR	EU5 CAT
A(H3N2)	29	34	9	25	12	21
A(H1N1pdm)	12	7	6	10	5	8
B/Yamagata	12	7	6	5	5	7
B/Victoria	12	8	6	5	5	7
All strains	23	23	6	25	13	18

EU5 (UK, DE, FR, ITA, and ESP) and VM (vaccine match).
